# Sudden Death during Parturition

**Published:** 1854-10

**Authors:** 


*Sudden Death duriny Parturition.*—Dr. CROOKE records, in a late number (Sept. 28, 1853) of the *Dublin Medical Press*, the following interesting case:—

"M. H., aged 36, was brought in a car to the Macroom Union Infirmary on the 3d inst. She stated that she was in labor of her sixth child; that her illness commenced two day previously, while traveling to join her husband, who had obtained employment in a distant part of the country; that she had not expected her confinement for another month; that she had been received into a farmer's house and kindly treated. Whilst answering my questions, she had a sharp pain, and, on examination, I found the os dilated to the size of a crown-piece, and very soft and yielding, a bag of membranes presenting, but no part of the fœtus was within reach. I should have conceived her to be not more than six hours in labor, were it not for her own statements, corroborated by the woman who accompanied her, to the effect that she had suffered occasional strong labor pains for forty-eight hours previously. She was a remarkably handsome, well-formed woman. Her circulation and respiration were good, and all the symptoms seemed to promise a safe if not a speedy delivery. I ordered a domestic enema, and left her in charge of a careful, intelligent nurse-tender, with directions to send for me when her labor was more advanced. In exactly an hour after, I was hastily summoned, and was at her bedside in ten minutes, but found she had expired in a few seconds after the message was sent to me. The nurse informed me that she had not left her for an instant; that her pains had not altered, either in character or frequency, until within a few minutes of the fatal termination; that then, during a strong pain, a small quantity of liquor amnii was discharged; that shortly after, a powerful expulsive effort followed, during which her face and neck became very livid; that, when the pain ceased, she complained that her heart was leaving her; that her respiration became suffocative, and she died in a few minutes. Having satisfied myself by auscultation that the fœtus was not living, I did not perform the Cæsarian section. I made a careful *post-mortem* examination in eighteen hours after. The body was well formed and moderately fat. The chest was very broad, and the mammary glands well developed. The face was pallid, and on the front of the neck there was considerable ecchymosis. The uterus was healthy; it contained a male fœtus of about seven months, very much macerated; the breech presented low down in the pelvis; there was a turn of the cord round the neck. The placenta was very firmly attached to the upper and anterior part of the fundus, and the usual quantity of liquor amnii was present. All the abdominal viscera were perfectly healthy. On opening the cavity of the chest, I found a quantity of fluid blood and some coagula, and I soon traced the source of it to be a rupture in the right pulmonary artery, just where it passes through the arch of the aorta. The heart and lungs were healthy, and the ruptured vessel did not indicate any proof of disease or weakness. Here, then, was the cause of death; it was altogether a fortuitous accident which no treatment could have averted; yet had this case occurred in private practice, where a *post-mortem* examination was not obtainable, the attending surgeon would have found it very difficult to absolve himself from blame, and the occurrence may have produced an injurious influence upon his practice for years."

